# Seasonal effects of wind conditions on migration patterns of soaring American white pelican

**DOI:** 10.1371/journal.pone.0186948

**Published:** 2017-10-24

**Authors:** Javier Gutierrez Illan, Guiming Wang, Fred L. Cunningham, D. Tommy King

**Affiliations:** 1 Department of Wildlife, Fisheries and Aquaculture, Mississippi State University, Starkville, United States of America; 2 Wildlife Services-National Wildlife Research Center, Mississippi Field Station, Mississippi State University, Starkville, United States of America; Hawaii Pacific University, UNITED STATES

## Abstract

Energy and time expenditures are determinants of bird migration strategies. Soaring birds have developed migration strategies to minimize these costs, optimizing the use of all the available resources to facilitate their displacement. We analysed the effects of different wind factors (tailwind, turbulence, vertical updrafts) on the migratory flying strategies adopted by 24 satellite-tracked American white pelicans (*Pelecanus erythrorhynchos*) throughout spring and autumn in North America. We hypothesize that different wind conditions encountered along migration routes between spring and autumn induce pelicans to adopt different flying strategies and use of these wind resources. Using quantile regression and fine-scale atmospheric data, we found that the pelicans optimized the use of available wind resources, flying faster and more direct routes in spring than in autumn. They actively selected tailwinds in both spring and autumn displacements but relied on available updrafts predominantly in their spring migration, when they needed to arrive at the breeding regions. These effects varied depending on the flying speed of the pelicans. We found significant directional correlations between the pelican migration flights and wind direction. In light of our results, we suggest plasticity of migratory flight strategies by pelicans is likely to enhance their ability to cope with the effects of ongoing climate change and the alteration of wind regimes. Here, we also demonstrate the usefulness and applicability of quantile regression techniques to investigate complex ecological processes such as variable effects of atmospheric conditions on soaring migration.

## Introduction

Understanding the factors driving species geographical distributions and seasonal movements is a central issue in ecology, especially in the context of global change [1]. However, disentangling the relative contributions of environmental factors is hampered by the confounding interactions of climate and other ecological variables [2]. Movements of flying migrants such as birds or insects are strongly influenced by the motion current in which the animals are immersed, forcing them to optimize risk-avoidance [3] as well as energy and time expenditure, especially regarding their flight altitudes and paths [4,5]. For that reason, animals that develop goal-oriented flying are expected to have evolved mechanisms for identifying and exploiting favourably-directed currents [6]. For example, animals may fly at times of the day and altitudes where the wind current is more supportive of their optimal direction [7]. Changing wind conditions have recently been suggested to affect not only bird and insect locomotion [8], but also foraging, reproductive success [9] and search for food resources and shelters [10]. Thus, migratory birds can select favourable (wind) conditions leading to faster, safer and more direct routes (time/energy-efficient) [11,12,13].

Some evidence exists about low geographical fidelity in migration routes (and stopover sites) of migratory birds between years [14] (but see [15]), suggesting that variable atmospheric conditions may be more determinant in the selection of migration strategies [16]. However, the underlying mechanisms used by free-flying birds for selecting these strategies remain unclear [17,18]. In particular, the flight of larger soaring birds is likely to be specially affected by atmospheric conditions, given that the higher cost of larger body mass displacement makes migration routes depend mainly on wind currents [19,20,21]. Optimization analyses of bird migration [22] with respect to the active selection of favourable winds, adaptation and orientation mechanisms in relation to wind conditions are challenging [23]. Many studies have demonstrated that high migratory activity often coincides with favourable winds (e.g., reviews by Richardson [24,25]). However, there is evidence in the literature for no selectivity of tailwind assistance in migration (Ospreys in Thorup *et al*. [26]; nocturnal passerine migrants in Alerstam *et al*. [27]) as well as of strong tailwind assistance (shorebirds in Green *et al*. [28]).

Flight generalists (i.e. birds that routinely switch between soaring and flapping flight (*sensu* Rayner [29]) use flapping when conditions are unsuitable for soaring [30,31]). By contrast, obligate soaring birds require updrafts to fly long distances because flapping flight is energetically constrained [32]. The use of thermal updrafts has been reported to be fundamental for migration of large species of birds over long distances, and these depend on vertical air motion to maintain their soaring and gliding flight [33,34]. These birds use convection or orographic lift to climb and gain potential energy and then glide to achieve energy-efficient soaring flight [35], generally avoiding large bodies of water where these wind currents are weaker [36]. After a gliding phase they need a new updraft or source of vertical air to climb again [37]. It is thus expectable that large soaring birds will search for vertical currents that appear to be physically necessary for their extended flight. Some existing evidence suggests that local and regional weather conditions exert a more decisive effect on soaring bird migration than large scale climate conditions [38]. However, empirical evidence of the effect of specific wind conditions on the flying strategy adopted by soaring birds is still scarce. Furthermore, it is still a challenging task to determine the effects of wind conditions [26] from other confounding factors such as cognitive flight/behaviour of other migrants [39] or favourable habitat selection [40], as well as the interpretation of these patterns.

Because early arrival times at breeding sites can be critically important for reproductive success, extended periods of unfavourable weather conditions (e.g. precipitation or extreme heat), while uncommon, can force birds to fly even when wind conditions are not optimal, e.g., when head-winds or updrafts are not available [41]. Birds may use different flying strategies between their spring and autumn migrations (see the case of Ospreys in Alerstam, Hake & Kjellen [42], Golden eagles in Miller *et al*. [10], Honey buzzards in Vansteelant *et al*. [19] and peregrine falcons in McGrady *et al*. [43]). Time may be a more important factor influencing migration strategies in spring than in autumn given the fitness benefits associated with an early arrival at the breeding destination [44]. It has also been argued that migration speeds in spring are greater than in autumn [43,10]. For these reasons, it seems plausible that spring migration could require more time and energy than autumn migration. Here, we test for differences in the migration strategies of soaring birds during spring and autumn in relation to wind conditions in the south-eastern United States, a topographically homogeneous region where the orographic features facilitate the model of effects of atmospheric conditions on bird migration.

### Target species

The mid-range migration of American white pelicans (*Pelecanus erythrorhynchos*) (hereafter AWPE) with their breeding in the central and northern ranges of North America and wintering regions in the southern US is a well-known phenomenon [5,45]. However, most of the information regarding AWPE migration routes results from analyses of band recoveries [46,47] and little information is available describing AWPE migration routes and corridors. Analyses of AWPE band recoveries show two major migratory corridors [46,48,49]. Pelicans from colonies west of the Rocky Mountains migrate to the Gulf of California and the Pacific coast of Mexico, while birds from east of the Continental Divide generally follow the Missouri and Mississippi River drainages to the Gulf of Mexico coast, Florida, and Central America. More recently satellite transmitter tagged AWPE from colonies in North and South Dakota followed a similar route [50]. In recent years, improvements in satellite tracking (including Global Positioning Systems [GPS] telemetry) in conjunction with Geographical Information Systems (GIS) have facilitated fine-scale analysis on the migration of soaring birds at individual levels [5]. Although such studies have been developed by monitoring these birds mainly by GPS tracking, an integrative approach to the mechanisms underlying the observed behaviours and migration strategies adopted by the AWPE is still lacking (but see [5]). Some empirical evidence of the behaviour of AWPE in their breeding geographical distribution is available but the behaviour within their wintering range has been far less studied [51,52]. Sovada et al [53] reported the advance of spring arrival of AWPE over the last decades presumably associated with enhanced fecundity due to early acquisition of prime breeding and foraging spots. However, earlier pelican arrival has also led to earlier nesting, with chicks being exposed to more days of severe weather during their most vulnerable period. Therefore, there is still much to debate about the balance of an early arrival and the frequent and severe losses of pelican chicks than might not have occurred if arrival and breeding on the colonies had not advanced. The main objective of this study is to analyse the relationship between model-derived atmospheric variables (wind conditions) and (hourly) migration speed (defined here as ground speed) and how these relationships may change during spring and autumn migration of AWPE populations along the Mississippi flyway. To accomplish this objective, we propose testing the following working hypotheses: (i) AWPE use vertical updrafts and favourable tailwinds more efficiently during the spring migration than during autumn in order to arrive at their breeding regions in the northern Great Plains; and (ii) AWPE fly faster and more direct routes during spring than in autumn as a response to the better physical condition in spring and the urgency to get to the breeding regions.

## Materials and methods

### Study system

**Fig 1:**
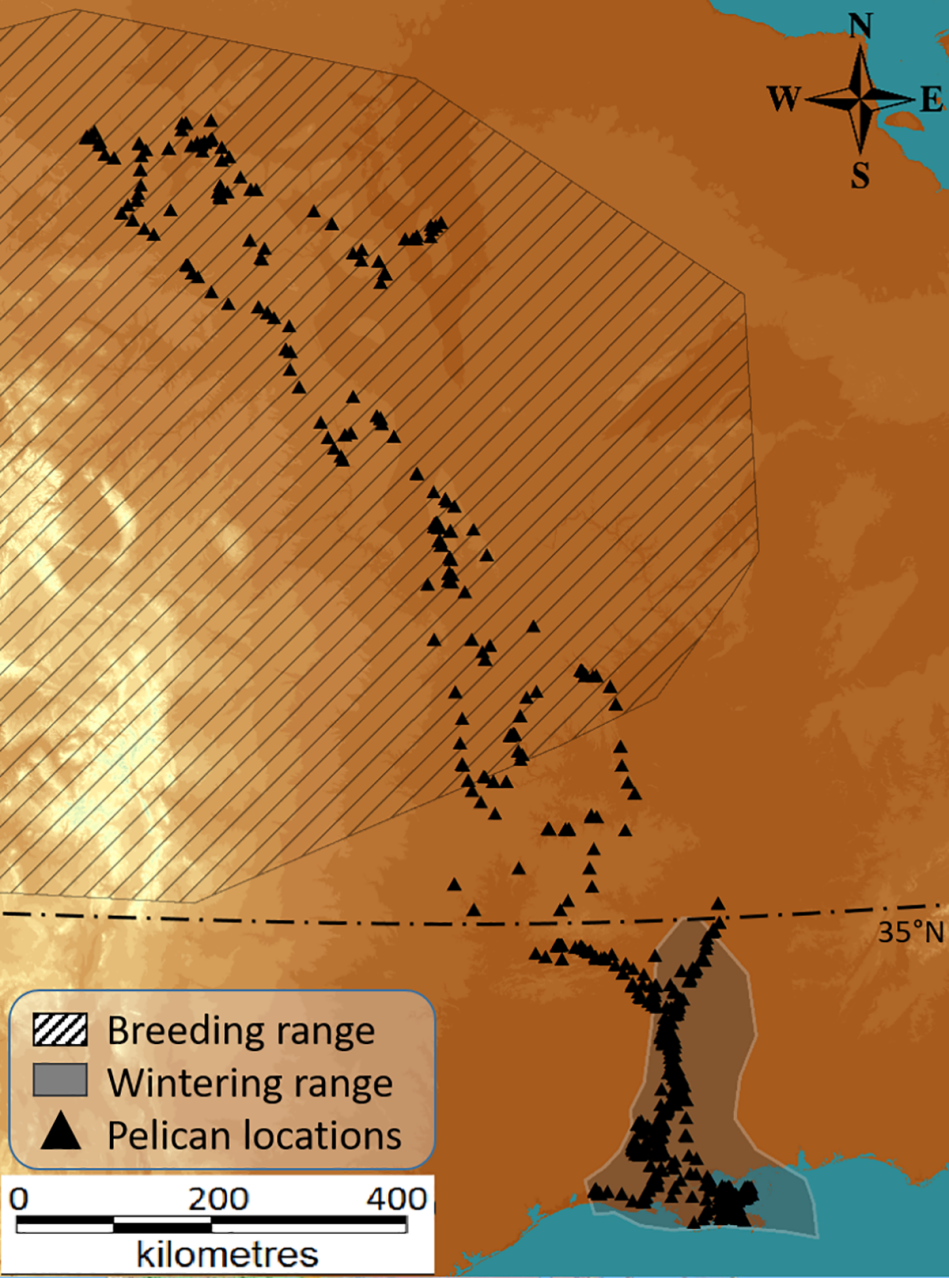
Map showing the geographical context of the study system, the breeding and wintering ranges of the target species and the migration routes for anexemplar individual of American white pelicans (AWPE). Elevation is shown in 100 m bands from <100 m (pale grey) to >1500 m (dark brown). Radio-tracked positions for an exemplar individual are shown as back triangles.

Our study system encompasses the corridor where migrating routes of AWPE populations occur (central flyway) both to and from their breeding range (Fig 1). This corridor is predominantly a flat region with elevations gradually increasing towards the North West. Largely dominant vegetation types are pastures, cultivated crops and deciduous forest [54].

### Bird data

We used data from AWPE tracked with satellite telemetry. Birds were captured from loafing sites during winter and spring at geographic regions within their wintering ranges [55]. They were equipped with a GPS transmitter that relocated the position of each bird hourly [52]. The complete data set comprised spatio-temporal locations of 24 birds that wintered in Alabama, Louisiana and Mississippi and had their breeding regions in the Northern Great Plains. These birds were tracked during one, two or a maximum of three years from 2002 to 2012 along variable migration routes. Due to weather conditions (mainly cloud cover that led to exhaustion of the solar battery), some locations at scheduled hours were not recorded and skipped to the next hour. To control for this problem, we only used segments between positions separated by 1 hour (which accounted for the 87% of location points). This dataset has provided high-resolution data on migratory movements of AWPE and represents a very rare and valuable source of information on their actual flying behaviour.

The spring and autumn migration were defined following King *et al*. [56]. A given individual was considered in spring migration (departure from wintering range) when it crossed 35°N latitudinal line northward (Fig 1), defined as the northern boundary of the wintering range. Spring migration ended when the given individual entered the breeding region and began moving locally as it searched or settled into a particular breeding site. Autumn migration was defined as the time from departure from summer range to the arrival into the wintering region. For the purpose of this study, we assume that pelicans soar rather than flap, and considered the “flapping” flight as non-significant during these displacements. This consideration is particularly important when determining the effects of environmental factors for soaring birds, whose movements rely primarily upon atmospheric conditions such as deflection updrafts and thermal convection as their primary means of gaining altitude for further displacement, whereas those birds using mostly flapping flight do not, in principle, depend on external forces as the main source of propulsion and lift [57].

We calculated the trajectory of each migration route, for each individual and each season using the GPS locations. The R package “adehabitatLT” [58] was then used to transform these coordinates into trajectories, a succession of locations of various lengths of movements and turn angles [59]. We then plotted in geographical context all the trajectories using ArcGIS (Environmental Systems Research Institute, Inc.). We analysed hourly segments of spring and autumn migration and calculated the average hourly flying speed during migration segments, which was used as a response variable in the models, following similar procedure as in Shamoun-Baranes *et al*. [60]. We also tested for differences in the cumulative speed, migration distance and duration leaving the breeding range to entering the wintering region or vice versa. Linear mixed models (LMMs) were built to detect differences in cumulative speed between spring and autumn migrations with animal identification being a random effect to account for temporal autocorrelation in the repeated measurements of the same individuals. If Akaike information criterion (AIC) of the LMM including season as a covariate was less than that of the null model with the intercept-only by 2.0, we concluded that migration speeds differed between spring and autumn. LMMs were implemented using the “nlme” package (https://cran.r-project.org/web/packages/nlme/index.html) [61].

### Wind data

We obtained wind vectors data from the North American Regional Reanalysis dataset via the “RNCEP” package [62]. This tool allows the access, organization, and visualization of weather data from the NCEP/NCAR and NCEP/DOE data sets. National Centre of Environmental Prediction (NCEP) Reanalysis data archives are provided by the NOAA/OAR/ESRL PSD, Boulder, Colorado, USA (http://www.esrl.noaa.gov/psd/). These are two long-term high-quality data sets with global coverage of many relevant atmospheric variables. Using GIS techniques, we extracted atmospheric data at each given GPS location/time of the AWPE via inverse distance weighting interpolation (IDW). These datasets have been used widely in studies of bird distributions [35,63]. We fit data for wind vectors at the 850 mb pressure level (roughly 1500 m.a.s.l.) that accounts for the boundary layer height in which the AWPE is expected to fly in its seasonal migration [5,64]. As a control measure, we also tested for the immediate upper (700 mb) and lower (925 mb) atmospheric layer and found no statistical differences in either the significance or the direction of the models (results not shown). The predictor variables included in the models were: Wind speed: u-wind (east-west component) measured in m/s; v-wind (South-North component) measured in m/s; ω-wind (Vertical velocity or omega component, proxy of vertical updrafts as described by Shannon *et al*. [5] measured in pa/s; TKE (Turbulent Kinetic Energy, proxy of atmospheric turbulence) measured in m^2^/s^2^. To control for potential multicollinearity, we evaluated correlations (Pearson’s r) between explanatory variables. Apart from these variables obtained from the NCEP database, we also calculated the tailwind component of the wind, relative to the direction of the pelican at each given location and time, measured in m/s (Table 1). We calculated the tailwind component (wind strength following the direction of migration) of the wind using the u and v vectors, the position of the pelican and applying basic trigonometry:

$$\mathrm{Tailwind}=\sqrt{(u^{2}+v^{2}}\;\cos\left(\left((atan2(u,v)(\frac{180}{\pi }))-angle)(\frac{\mu }{180}\right)\right).$$

Wind direction (wd) at a location was computed using the formula wd = atan2 (u, v) in the R environment. The interaction of wind speed and direction becomes a critical measure for interpreting the appropriate effects of wind on pelican flight. Therefore, to test for the relationship between the flight direction of each bird and direction of the wind at each given location, we conducted circular statistics correlation between the track direction of outward flights and wind direction patterns. Significance of tests was assessed by a circular version of the Pearson’s correlation. Statistical procedures were performed with ‘‘circular” package (https://cran.r-project.org/web/packages/circular/index.html) [65].

**Table 1:** List of environmental variables included in quantile regression of hourly speed of American white pelicans (AWPE).

Atmospheric variable	Code	Mean (min-max) Spring	Mean (min-max) Autumn	Units
U-Wind Component (East to West)	u	5.36 (-8.96–22.38)	4.83 (-12.39–23.17)	m/s
V-Wind Component (South to North)	v	3.02 (-17.85–23.24)	-0.21 (-20.01–25.16)	m/s
Omega (Vertical Velocity)	ω	-0.01 (-0.48–0.45)	0.03 (-0.45–0.32)	Pascal/s
Tailwind component (flight direction)	tailw	0.33 (-26.96–25.52)	-0.21 (-22.76–25.53)	m/s
Turbulent Kinetic Energy	tke	62.18 (0.02–391.57)	50.32 (0.04–411.28)	m^2^/s^2^
**Response variable**	Code	Mean (min-max) Spring	Mean (min-max) Autumn	Units
Average migration speed	speed	1.32 (0–81.39)	1.11 (0–53.28)	m/s

### Model development and statistical analysis

Spring and autumn migration were analysed separately. We generated quantile regression models using the “quantreg” package (https://cran.rproject.org/web/packages/quantreg/index.html) [66]. When we are not only interested in estimating rates of change in the mean of the response variable conditional on the levels or values of independent variables, quantile regression is an appropriate way to estimate the conditional quantiles of a response variable distribution [67]. This analysis provides a more complete view of possible causal relationships between variables in ecological processes [68,69]. Furthermore, quantile regression is insensitive to heteroscedastic errors and dependent variable outliers [70], both of which significantly affect least squares models [71]. Birds carrying out active and directional migration fly at higher altitudes and a faster speed than those engaged in local movements [72]. Primary migration movements were identified by carrying out quantile regressions focusing on the high quantiles of our response variable (distance travelled per hour). We estimated a series of quantile regression from the 50^th^ to the 95^th^ quantile. To generate the best model for each quantile, we performed a stepwise backward model selection. Starting with a full model (more conservative procedure), at each step, one term was deleted following p-value significance (p<0.05 for variable inclusion, p>0.10 for removal), until no additional deletion of terms improved the model.

## Results

Based on the full dataset with the 24 radio-tracked individuals, we calculated the trajectory for each bird by season, resulting in a total of 80,360 hourly migration segments. Mean hourly speed during the migration was 39.53 km/h (spring) and 33.16 km/h (autumn). Cumulative speed was significantly greater in spring than in autumn (AIC-null = 290.53, AIC—season = 280.87, ΔAIC = 9.66; coefficient for spring = 3.65). Cumulative migration distance tended to be shorter in spring than in autumn (AIC-null = 709.04, AIC-season = 699.52, and ΔAIC = 9.52; coefficient for spring = -51.04).

With regard to the circular statistical analyses we found that the correlation between flight direction of AWPE and wind direction was progressively more significant while flying at greater speeds (in higher quantiles). During both spring and autumn, the correlation was significant (*p* < 0.05) for flying speeds above 10 km/h (Table 2).

**Table 2:** Circular correlation coefficients between the flying direction of the American white pelicans and the encountered wind direction at each given recorded location. Results are shown for each season using the complete dataset and three different speed thresholds.

Spring	N	Correlation	Test statistic	p-value
Complete data	2320	0.026	1.277	0.20
Speed > 5 km/h	182	0.171	2.213	0.06
Speed > 10 km/h	95	0.206	1.942	<0.05
Speed > 12 km/h	53	0.335	2.431	<0.01
**Autumn**				
Complete data	4093	-0.011	-0.719	0.47
Speed > 5 km/h	394	0.050	0.948	0.34
Speed > 10 km/h	189	0.205	2.569	0.01
Speed > 12 km/h	129	0.260	2.688	<0.01

**Fig 2:**
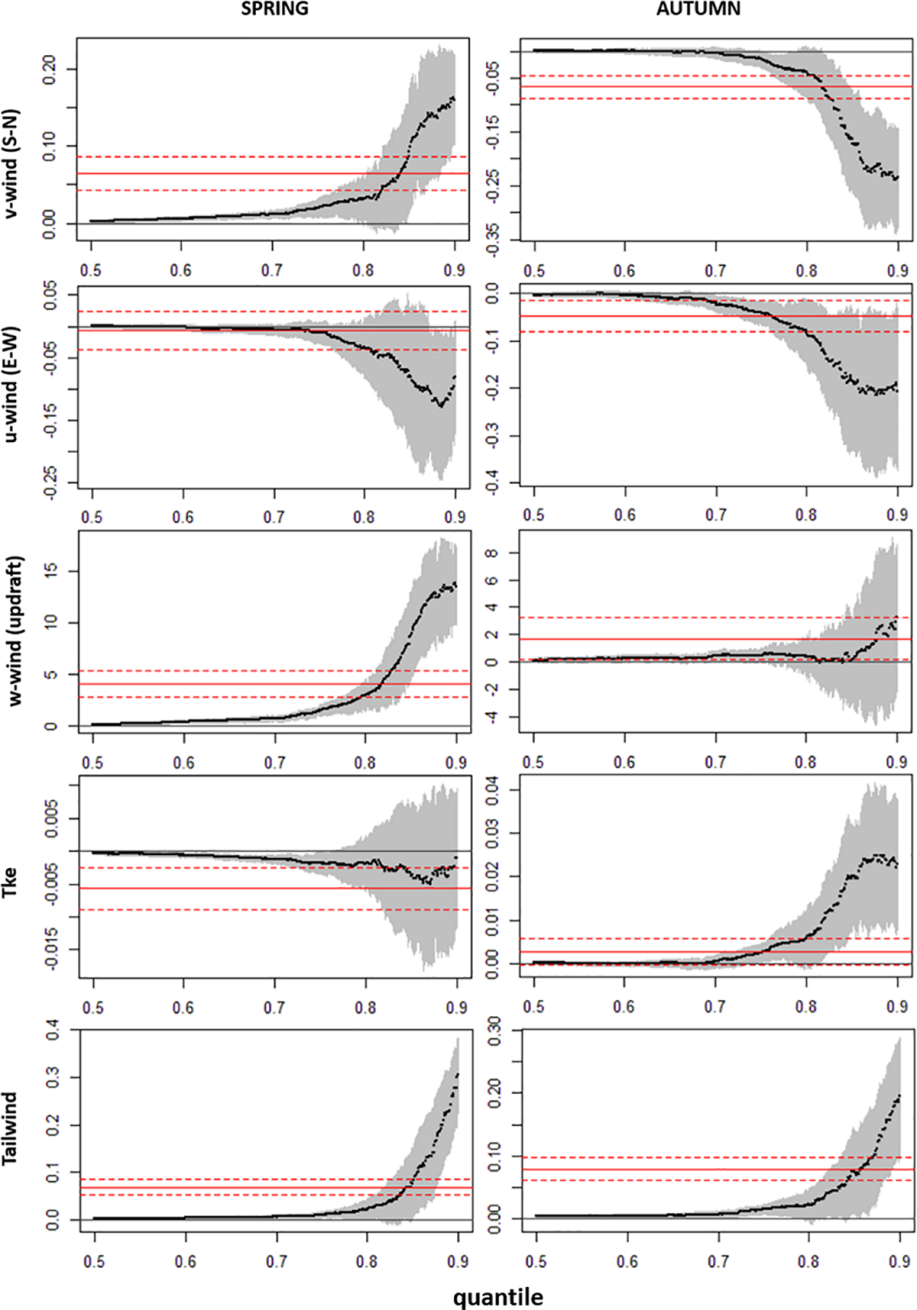
Results of the quantile regression analyses of the hourly flying speed of AWPE for each season and each of the environmental predictors considered. Each black dot is the slope coefficient for the quantile indicated on the x axis. The red lines are the least squares estimate (solid) and its 95% confidence interval (dashed). The grey area represents the 95% confidence interval for the quantile regression estimates plotted as the black line.

Prior to the model development, we tested for inter-correlations between the predictor variables (S1 Table). Out of the final set of variables selected for analysis we found none that were highly correlated (all r_p_ < |0.7|). The final set of explanatory environmental variables is listed in Table 1. We found highly consistent results for each of the predictor variables in the quantile regression (Fig 2, S2 Table). In the case of v-wind, it was selected for all quantile regression models in spring and all the models above the 70^th^ quantile in autumn. For models between the 70^th^ and 95^th^ quantiles, it became progressively more positive and significantly different from zero during spring migration, and progressively more negative and significantly different from zero during autumn (Fig 2, S3 Table). The tailwind component was selected in all the models for both spring and autumn (S2 Table), and for quantile regressions above the 60th quantile, its positive effect progressively increased and became more significantly different from zero during both spring and autumn migration (Fig 2). While vertical velocity (ω, available vertical uplift) had no effect during the autumn migration for the higher quantiles (above the 70^th^ quantile), it was selected in all the models and its effect was consistently and progressively more positive and significantly different from zero during spring migration. In all the cases for v-wind, tailwind and the omega parameter (ω) during spring, the quantile regression results were significantly different from those of the ordinary least squares (OLS) regression, with lower and upper quantiles well beyond the average conditions described by least squares estimates (Fig 2, red lines). In autumn, the results show that the OLS regression slope was sufficient to describe the relationship between x and y (the quantile slope estimates are not statistically different from the OLS estimate). We also found mixed results regarding the effects of the west-east wind component (u-wind) and the turbulence kinetic energy (tke). We found a negative effect of the u-wind component during both spring and autumn migration. These results appear to contradict the “loop migration” theory, which propose that migratory birds will follow different routes in spring and autumn, taking advantage of the predominant east or west winds. Finally, we found an increasingly positive effect of the kinetic turbulence during the autumn migration, but no significant effect during spring migration.

## Discussion

Animals migrate in response to external and internal factors, among which, meteorological conditions are expected to play a crucial role in their displacements [22,73]. As discussed by Chapman *et al*. [7], the conditions of the moving medium (e.g. water, air) affect the strategy that animals adopt in order to accomplish their movements. Depending on their size and physical condition, migrating birds may select the optimal energy expenditures, adapting their movements following two non-mutually exclusive types of response: they can simply be transported downstream passively or actively try to compensate for any drift away from their marked goal within the flow. We expected that AWPE would be able to adopt different migratory strategies depending on the wind conditions, in which they can simply drift or compensate if the wind conditions are not as favourable and they are physically capable of doing so [74]. Several studies have documented that many species take advantage of winds following their flight direction during migration [10,25]. However, to our knowledge, this is the first fine-scale study that investigates how the contribution of wind factors changes depending on the migration speed of large soaring birds.

Our GPS-tracked AWPE data allowed us to record and analyse their autumn and spring migration routes, including hourly speed and cumulative speed. Three factors are arguably the main drivers of the seasonal variability in migration speed (at least for adult individuals): (i) physical condition of birds; (ii) wind assistance/opposition; and (iii) vertical updrafts/convection. Evidence exists for both faster spring [10,43] but also for faster autumn migration [60], as well as for different timing regarding stopovers in these routes [42]. We found faster hourly migration speeds in spring (39.53 km/h) than in autumn (33.16 km/h), that were on average reasonably close to those obtained by Leshem & Yom-Tov [75] on great white pelicans (*Pelecanus onocrotalus)* in Israel (42.5 km/h). We also found significantly faster cumulative speed in spring. Specifically, all the individuals showed greater cumulative speed during the spring migration, and 21 of the 22 migration routes were flown at greater speeds in spring than in their counterpart autumn migration for the same individual. This is consistent with the tendency to minimize migration time and the hypothesis of arriving at the breeding regions achieved by the individuals in prime condition [76], leading pelicans to fly faster and more direct routes in spring than in autumn (see example with Golden Eagles in Miller *et al*. [10]). It was shown by Mandel *et al*. [73] that, unlike flapping, soaring flight does not lead to greatly increased metabolic costs. Watanabe [77] recently reported larger migration ranges and low flight costs irrespective of body mass in soaring birds in comparison with flapping birds. In this same study, it was found that turkey vultures (*Cathartes aura*) depend on high levels of kinetic turbulence and thermal updrafts to achieve longer flight distances (see also [78,79]). Therefore, it is expected that atmospheric conditions (favourable winds, turbulence, and presence of thermal updrafts) are the main driving factors of soaring migration, affecting their routes and time expenditure during the journey.

Several species of large birds, including pelicans, have been found to fly at higher altitudes to gain potential energy and achieve higher speeds and better soaring conditions for gliding [65,75]. When soaring birds glide continuously in straight horizontal line they gain little or no altitude, relying on the presence of updrafts, but benefit from increased speed cross-country, optimizing the migration duration [39,80]. Thus, they need to find a balance between straight-line gliding and discrete thermal lift [81] to optimize time and energy expenditure. Our results support this theory since, during the spring migration, soaring flight was faster and more direct (better physical condition, hence more compensation), and was strongly supported by the presence of updrafts (Fig 2). AWPE thus seem to follow different flying strategies during spring and autumn, actively selecting updrafts during northward spring migration that are not available to the birds when travelling southward in autumn, but relying heavily on the tailwinds during their slower autumn migration (more drifting). This may be due to poorer physical condition, gliding from the higher northern plains and taking advantage of the potential energy. This leads us to believe that they need to adjust their flying strategy during spring when birds are pressured by the urgency to arrive at the breeding regions as soon as possible. This could have implications in the facultative migrant behaviour of some immature individuals, and explain why some young AWPE can often migrate southward, but they do not migrate northward during their first spring.

It has been argued that AWPE attempt to use the strongest and widest updrafts during the peak time of the day for the thermal initiation, and then, soar as long as the conditions (predominantly lift and tailwind) permit [5]. In our study, AWPE predominantly flew during the hours of the day when the thermal convection generation is at its peak. We did not find a difference in the time of flight between spring and autumn.

Strong tailwind assistance is used by many shorebirds (e.g. [28], but see Results in [26]). Quantile regression results reported here are consistent with the hypothesis of favourable wind currents facilitating the migration in both seasons, as tailwind effect progressively increased in both spring and autumn (greater in higher quantiles) The circular statistical analyses on the seasonal direction of the wind confirmed our expectations that wind coming from the south facilitates the spring migration of AWPE, and winds from the north facilitate autumn migration. Finally, there are a few well-known cases of loop migration in the literature [82,83], that address the benefits of changing the migration routes between seasons and taking advantage of the optimal west or east wind currents. According to these studies, the energy-saving benefits appear to outweigh the drawbacks of finding alternative routes and stopover sites, and it may in fact be a rule rather than exception [17]. However, even though we did find some indications that this must be the case for some individuals, we did not find sufficient evidence of AWPE using predominantly looping main routes on autumn and spring migration.

Our analyses demonstrate the usefulness of quantile regression for statistical inference of ecologically complex distributions such as those in our study. Results presented here show that, at increasingly greater speeds, favourable wind components exert an increasingly more supportive effect on the soaring flight, an effect that we would have not been detected using an ordinary least squares modelling approach. For example, quantile regression analysis indicated that the trend for increasing flying speed with increasing (spring) or decreasing (autumn) v-wind component was stronger for the locations with greater flying speed, and more significant above the 70^th^ quantile (Fig 2). While our results regarding the supportive effect (both strength and direction) of favourable winds (tailwind and v-wind in opposite direction in spring and autumn, and *w* in spring) make complete biological sense and might be even expectable, to our knowledge it has never been shown before, particularly at this fine scale and empirically based on actual tracking data. As shown by Mandel *et al*. [73], some species of soaring birds can benefit from turbulent air conditions in-flight. This might particularly important when development of thermal updrafts is poor [84]. Although we did not find conclusive evidence to support these previous findings in other species, we did find seasonal effects of turbulence (tke) in our models, significantly positive in autumn, but not in spring. The fact that we found a positive effect of tke in autumn suggests that these theoretically unfavourable wind conditions do not hinder the migration of AWPE, but seem to facilitate their flight at least in this section of their journey. These results may seem in agreement with Mallon, Bildstein & Katzner [84], in which turbulence was shown to be beneficial for two species of large soaring birds. However, it is worth noting that these fine-scale eddies (sensu [84]) are unlikely to be exploited by a large soaring bird such as the AWPE. The patterns we found could be partially explained by the fact that birds are in poorer condition during the autumn migration after the breeding season [85,86], which could lead to pelicans taking advantage of the lift generated by turbulence encountered during their flight, as an energy-saving mechanism. Additionally, the higher elevation at northern latitudes provides a source of potential energy, so that the lift by turbulences may be sufficient for gliding down southward during the autumn migration.

## Conclusions

The results from this study reveal that wind conditions play an important role in AWPE migration in North America. Thus, wind-based models could be an appropriate tool for making forecasts on migratory behaviour and range shifts based on climate change scenarios at fine resolutions and regional scales. Our results suggest that pelicans might be forced to maximize the use of wind resources, including favourable tailwinds and supportive updrafts during spring migration due to the competition for early arrival in the breeding regions. Pelicans were able to use wind resources differently in spring and in autumn, resulting in faster migration when flying towards the breeding regions. This flexibility in the migration strategy of the AWPE, facilitated by their ability to use energetically efficient soaring, suggests that our target species is likely to cope with ongoing climate change that is expected to alter winds regimes and the energy landscape (*sensu* Shepard *et al*. [87]) in the near future. Using quantile regression techniques we were able to detect variable wind effects on the flying speed that would not have been detected with an ordinary least squares approach. Improvements in telemetry technologies and behavioural modelling have greatly enhanced migration research. Nevertheless, continuous habitat protection may be essential to facilitate migration routes, given the threat of discontinuous migration corridors if key stopover sites are affected by land use change or fragmentation of habitat currently used by these pelicans (e.g. Mississippi alluvial valley) [43,74]. Furthermore, precise evaluation of the role of further variables (other than wind conditions) concerning biotic interactions and land cover may be crucial to assess the applicability of the models.

## Supporting information

S1 Table The Pearson correlations (Pearson’s r) coefficients among the predictor variables used in the analyses of the hourly flying speed of American white pelicans. We also include the response variable in the table.
(PDF)

S2 Table Results of the stepwise model selection showing the variables selected (+ and–symbols denote positive and negative effects, respectively) in the final models of hourly flying speed of American white pelicans (AWPE) for each quantile. Acronyms stand for u-wind (u); v-wind (v); vertical velocity (v), turbulent kinetic energy (tke) and tailwind (tailw).
(PDF)

S3 Table Backward stepwise model selection process for the hourly flying speed of American white pelicans (p>0.1 as threshold to remove term). (PDF)
