# Supplementary material for: Seasonal effects of wind conditions on migration patterns of soaring American white pelican
Source: PLoS One. 2017 Oct 24;12(10):e0186948. doi: 10.1371/journal.pone.0186948 (PMC5655449; doi:10.1371/journal.pone.0186948)
Supplement: S1 Table — We also include the response variable in the table. (PDF) [file pone.0186948.s001.pdf]

Spring:

	**u-wind**	**v-wind**	**w (uplift)**	**tke**	**tailwind**	**speed**
**u-wind**	1.000	0.188	-0.243	0.651	-0.046	-0.069
**v-wind**	0.188	1.000	-0.533	0.493	0.072	0.025
**w (uplift)**	-0.243	-0.533	1.000	-0.352	-0.034	0.071
**tke**	0.651	0.493	-0.352	1.000	-0.019	-0.068
**tailwind**	-0.046	0.072	-0.034	-0.019	1.000	0.128
**speed**	-0.069	0.025	0.071	-0.068	0.128	1.000

Autumn:

	**u-wind**	**v-wind**	**w (uplift)**	**tke**	**tailwind**	**speed**
**u-wind**	1.000	0.294	-0.204	0.653	0.011	-0.092
**v-wind**	0.294	1.000	-0.472	0.287	-0.065	-0.178
**w (uplift)**	-0.204	-0.472	1.000	-0.085	0.037	0.129
**tke**	0.653	0.287	-0.085	1.000	0.061	-0.038
**tailwind**	0.011	-0.065	0.037	0.061	1.000	0.176
**speed**	-0.092	-0.178	0.129	-0.038	0.176	1.000
