# Supplementary material for: Seasonal effects of wind conditions on migration patterns of soaring American white pelican
Source: PLoS One. 2017 Oct 24;12(10):e0186948. doi: 10.1371/journal.pone.0186948 (PMC5655449; doi:10.1371/journal.pone.0186948)
Supplement: S2 Table — Acronyms stand for u-wind (u); v-wind (v); vertical velocity (v), turbulent kinetic energy (tke) and tailwind (tailw). (PDF) [file pone.0186948.s002.pdf]

	Spring					autumn				
**Quantiles**	**u**	**v**	**ω**	**tke**	**tailw**	**u**	**v**	**ω**	**tke**	**tailw**
**0.5**		+	+	-	+			+		+
**0.6**		+	+	-	+			+		+
**0.7**		+	+	-	+	-		+		+
**0.8**		+	+	-	+	-	-			+
**0.9**		+	+		+	-	-		+	+
**0.95**		+	+		+	-	-		+	+
**0.99**		+	+		+	-	-		+	+
