# Supplementary material for: Seasonal effects of wind conditions on migration patterns of soaring American white pelican
Source: PLoS One. 2017 Oct 24;12(10):e0186948. doi: 10.1371/journal.pone.0186948 (PMC5655449; doi:10.1371/journal.pone.0186948)
Supplement: S3 Table — (PDF) [file pone.0186948.s003.pdf]

**Spring**

```
Step 1

tau: [1] 0.5

Coefficients:
              Value     Std. Error t value   Pr(>|t|)
(Intercept)   0.11998   0.01055    11.36815  0.00000
v             0.00282   0.00092     3.05832  0.00224
u             0.00052   0.00157     0.33108  0.74060
w             0.14382   0.05386     2.67012  0.00762
tke          -0.00037   0.00010    -3.80486  0.00014
tailw         0.00147   0.00055     2.65079  0.00807

Remove term: u-wind


tau: [1] 0.6

Coefficients:
              Value     Std. Error t value   Pr(>|t|)
(Intercept)   0.25790   0.02147    12.01126  0.00000
v             0.00571   0.00174     3.28752  0.00102
u            -0.00154   0.00305    -0.50451  0.61394
w             0.39397   0.10882     3.62024  0.00030
tke          -0.00060   0.00022    -2.76671  0.00569
tailw         0.00387   0.00117     3.30853  0.00095

Remove term: u-wind


tau: [1] 0.7

Coefficients:
              Value     Std. Error t value   Pr(>|t|)
(Intercept)   0.53197   0.04393    12.10862  0.00000
v             0.01174   0.00301     3.89596  0.00010
u            -0.00568   0.00447    -1.27087  0.20386
w             0.70889   0.16449     4.30964  0.00002
tke          -0.00127   0.00036    -3.49559  0.00048
tailw         0.00631   0.00207     3.04400  0.00235

Remove term: u-wind
```

```
tau: [1] 0.8

Coefficients:
             Value    Std. Error t value  Pr(>|t|)
(Intercept)  0.83390  0.08790     9.48699  0.00000
v            0.01944  0.00753     2.58339  0.00983
u           -0.01030  0.01025    -1.00434  0.31529
w            1.46817  0.35236     4.16663  0.00003
tke         -0.00180  0.00096    -1.87647  0.06068
tailw        0.01115  0.00425     2.62450  0.00872

Remove term: u-wind


tau: [1] 0.9

Coefficients:
             Value    Std. Error t value  Pr(>|t|)
(Intercept)  7.47706  0.36489    20.49105  0.00000
v            0.15990  0.03505     4.56240  0.00001
u           -0.08045  0.05369    -1.49831  0.13415
w           13.50350  2.30317     5.86301  0.00000
tke         -0.00106  0.00638    -0.16548  0.86858
tailw        0.30477  0.04831     6.30914  0.00000

Remove term: tke


tau: [1] 0.95

Coefficients:
             Value    Std. Error t value  Pr(>|t|)
(Intercept)  9.69862  0.31464    30.82440  0.00000
v            0.13554  0.03421     3.96219  0.00008
u           -0.00093  0.04526    -0.02057  0.98359
w           13.52857  1.89109     7.15386  0.00000
tke         -0.00016  0.00533    -0.02946  0.97650
tailw        0.40949  0.03507    11.67623  0.00000

Remove term: u-wind


tau: [1] 0.99

Coefficients:
             Value    Std. Error t value  Pr(>|t|)
(Intercept) 13.38660  0.43156    31.01913  0.00000
v            0.13702  0.03845     3.56381  0.00037
u            0.11876  0.04569     2.59930  0.00938
w            8.15756  2.25670     3.61483  0.00031
tke         -0.00678  0.00598    -1.13293  0.25733
tailw        0.57450  0.01883    30.50216  0.00000

Remove term: tke
```

Step 2

```
tau: [1] 0.5

Coefficients:
            Value    Std. Error t value  Pr(>|t|)
(Intercept)  0.12103  0.00992   12.19469  0.00000
v            0.00275  0.00082    3.34371  0.00084
w            0.13996  0.05773    2.42429  0.01539
tke         -0.00035  0.00007   -5.20544  0.00000
tailw        0.00144  0.00066    2.19230  0.02843

Final model: v+w+tke+tailw


tau: [1] 0.6

Coefficients:
            Value    Std. Error t value  Pr(>|t|)
(Intercept)  0.25450  0.01910   13.32335  0.00000
v            0.00597  0.00156    3.81322  0.00014
w            0.39978  0.11068    3.61206  0.00031
tke         -0.00068  0.00014   -5.01042  0.00000
tailw        0.00402  0.00121    3.32001  0.00091

Final model: v+w+tke+tailw


tau: [1] 0.7

Coefficients:
            Value    Std. Error t value  Pr(>|t|)
(Intercept)  0.51746  0.03788   13.66150  0.00000
v            0.01178  0.00250    4.71926  0.00000
w            0.73038  0.18900    3.86444  0.00011
tke         -0.00156  0.00023   -6.72616  0.00000
tailw        0.00649  0.00215    3.02567  0.00250

Final model: v+w+tke+tailw


tau: [1] 0.8

Coefficients:
            Value    Std. Error t value  Pr(>|t|)
(Intercept)  0.80915  0.08020   10.08875  0.00000
v            0.02118  0.00597    3.54897  0.00039
w            1.45053  0.29796    4.86827  0.00000
tke         -0.00247  0.00067   -3.71188  0.00021
tailw        0.00972  0.00466    2.08630  0.03703

Final model: v+w+tke+tailw
```

tau: [1] 0.9

```
Coefficients:
            Value    Std. Error t value  Pr(>|t|)
(Intercept)  7.41981  0.35389   20.96654  0.00000
u           -0.08581  0.04940   -1.73689  0.10250
v            0.14972  0.03372    4.44075  0.00001
w           13.15647  2.44914    5.37187  0.00000
tailw        0.30672  0.03874    7.91834  0.00000
```

Remove term: u-wind

tau: [1] 0.95

```
Coefficients:
            Value    Std. Error t value  Pr(>|t|)
(Intercept)  9.69525  0.32342   29.97705  0.00000
v            0.13583  0.03008    4.51504  0.00001
w           13.56846  1.63827    8.28219  0.00000
tke         -0.00027  0.00453   -0.06071  0.95159
tailw        0.40841  0.03587   11.38574  0.00000
```

Remove term: tke

tau: [1] 0.99

```
Coefficients:
            Value    Std. Error t value  Pr(>|t|)
(Intercept) 13.31426  0.42748   31.14581  0.00000
u            0.06530  0.05161    1.26532  0.20585
v            0.09688  0.04028    2.40545  0.01621
w            9.07035  2.73120    3.32101  0.00091
tailw        0.58460  0.03350   17.45015  0.00000
```

Remove term: u-wind

Step 3

tau: [1] 0.9

```
Coefficients:
            Value    Std. Error t value  Pr(>|t|)
(Intercept)  7.18570  0.35436   20.27807  0.00000
v            0.14329  0.03785    3.78604  0.00016
w           13.67052  2.66104    5.13728  0.00000
tailw        0.33060  0.03451    9.57961  0.00000
```

Final model: v+w+tailw

```
tau: [1] 0.95

Coefficients:
            Value    Std. Error t value  Pr(>|t|)
(Intercept)  9.69850  0.28467   34.06926  0.00000
v            0.13506  0.02632    5.13091  0.00000
w           13.44514  2.15991    6.22486  0.00000
tailw        0.41231  0.02455   16.79266  0.00000

Final model: v+w+tailw


tau: [1] 0.99

Coefficients:
            Value    Std. Error t value  Pr(>|t|)
(Intercept) 13.69935  0.37778   36.26270  0.00000
v            0.09874  0.05035    1.96102  0.04996
w            8.10047  3.46732    2.33623  0.01954
tailw        0.55793  0.04173   13.36949  0.00000

Final model: v+w+tailw
```

## Results

```
MODELS (by quantile):

0.5: speed ~ v + w + tke + tailw
0.6: speed ~ v + w + tke + tailw
0.7: speed ~ v + w + tke + tailw
0.8: speed ~ v + w + tke + tailw
0.9: speed ~ v + w + tailw
0.95: speed ~ v + w + tailw
0.99: speed ~ v + w + tailw
```

## Autumn

Step 1

tau: [1] 0.5

Coefficients:

| | Value | Std. Error | t value | Pr(>\|t\|) |
|---|---|---|---|---|
| (Intercept) | 0.10827 | 0.01595 | 6.78712 | 0.00000 |
| v | 0.00060 | 0.00161 | 0.37081 | 0.71082 |
| u | -0.00295 | 0.00245 | -1.20628 | 0.22786 |
| w | 0.15696 | 0.09107 | 1.72346 | 0.08497 |
| tke | 0.00015 | 0.00020 | 0.74575 | 0.45591 |
| tailw | 0.00408 | 0.00122 | 3.33062 | 0.00088 |

Remove term: v-wind

tau: [1] 0.6

Coefficients:

| | Value | Std. Error | t value | Pr(>\|t\|) |
|---|---|---|---|---|
| (Intercept) | 0.25090 | 0.02853 | 8.79494 | 0.00000 |
| v | 0.00010 | 0.00280 | 0.03698 | 0.97050 |
| u | -0.00359 | 0.00470 | -0.76356 | 0.44522 |
| w | 0.26301 | 0.16876 | 1.55852 | 0.11928 |
| tke | -0.00014 | 0.00036 | -0.38220 | 0.70235 |
| tailw | 0.00522 | 0.00168 | 3.10389 | 0.00194 |

Remove term: v-wind

tau: [1] 0.70

Coefficients:

| | Value | Std. Error | t value | Pr(>\|t\|) |
|---|---|---|---|---|
| (Intercept) | 0.73024 | 0.08442 | 8.64987 | 0.00000 |
| v | -0.01555 | 0.00959 | -1.62052 | 0.10529 |
| u | -0.04065 | 0.01419 | -2.86382 | 0.00423 |
| w | 0.48705 | 0.26037 | 1.87059 | 0.06156 |
| tke | 0.00236 | 0.00122 | 1.93000 | 0.05376 |
| tailw | 0.01594 | 0.00464 | 3.43595 | 0.00060 |

Remove term: v-wind

```
tau: [1] 0.80

Coefficients:
            Value    Std. Error t value  Pr(>|t|)
(Intercept)  0.87418  0.12793    6.83356  0.00000
v           -0.03054  0.01386   -2.20301  0.02771
u           -0.06054  0.02113   -2.86476  0.00422
w            0.45322  0.35364    1.28161  0.20014
tke          0.00442  0.00198    2.23231  0.02571
tailw        0.01896  0.00759    2.49738  0.01260

Remove term: w


tau: [1] 0.9

Coefficients:
            Value    Std. Error t value  Pr(>|t|)
(Intercept)  5.08166  0.55688    9.12525  0.00000
v           -0.23565  0.05611   -4.19978  0.00003
u           -0.20733  0.10154   -2.04182  0.04131
w            3.21559  3.26008    0.98635  0.32408
tke          0.02301  0.00904    2.54419  0.01103
tailw        0.19441  0.05739    3.38727  0.00072

Remove term: w


tau: [1] 0.95

Coefficients:
            Value    Std. Error t value  Pr(>|t|)
(Intercept)  8.53905  0.41443   20.60411  0.00000
v           -0.15315  0.03906   -3.92086  0.00009
u           -0.23225  0.05060   -4.58964  0.00000
w            2.37924  2.97881    0.79872  0.42455
tke          0.02218  0.00657    3.37382  0.00076
tailw        0.39535  0.04906    8.05847  0.00000

Remove term: w


tau: [1] 0.99

Coefficients:
            Value    Std. Error t value  Pr(>|t|)
(Intercept) 12.43838  0.96798   12.84990  0.00000
v           -0.09561  0.06013   -1.59013  0.11197
u           -0.10694  0.07211   -1.48300  0.13824
w           -1.28315  4.03990   -0.31762  0.75081
tke          0.00568  0.01070    0.53025  0.59600
tailw        0.47259  0.04918    9.61033  0.00000

Remove term: w
```

Step 2

```
tau: [1] 0.5

Coefficients:
            Value    Std. Error t value  Pr(>|t|)
(Intercept)  0.10961  0.01531    7.15808  0.00000
u           -0.00310  0.00242   -1.28352  0.19946
w            0.14256  0.09666    1.47489  0.14041
tke          0.00017  0.00018    0.91849  0.35848
tailw        0.00398  0.00119    3.35146  0.00082

Remove term: tke


tau: [1] 0.6

Coefficients:
            Value    Std. Error t value  Pr(>|t|)
(Intercept)  0.25203  0.02539    9.92673  0.00000
u           -0.00363  0.00323   -1.12601  0.26031
w            0.26882  0.10816    2.48539  0.01303
tke         -0.00014  0.00024   -0.55635  0.57803
tailw        0.00515  0.00141    3.64011  0.00028

Remove term: tke


tau: [1] 0.7

Coefficients:
            Value    Std. Error t value  Pr(>|t|)
(Intercept)  0.52143  0.05222    9.98562  0.00000
u           -0.01688  0.00992   -1.70174  0.08897
w            0.50521  0.28148    1.79484  0.07284
tke          0.00000  0.00081    0.00197  0.99843
tailw        0.00785  0.00385    2.03850  0.04164

Remove term: tke


tau: [1] 0.8

Coefficients:
            Value    Std. Error t value  Pr(>|t|)
(Intercept)  1.17867  0.20797    5.66742  0.00000
u           -0.09131  0.03897   -2.34308  0.01923
v           -0.04719  0.02640   -1.78748  0.07402
tke          0.00636  0.00425    1.49680  0.15461
tailw        0.02203  0.01564    1.40824  0.13922

Remove term: tke
```

```
tau: [1] 0.9

Coefficients:
            Value    Std. Error t value  Pr(>|t|)
(Intercept)  5.08934  0.64573    7.88152  0.00000
u           -0.22383  0.10700   -2.09184  0.03658
v           -0.25094  0.05935   -4.22809  0.00002
tke          0.02526  0.01013    2.49416  0.01271
tailw        0.19453  0.05760    3.37706  0.00075

Final model: u+v+tke+tailw


tau: [1] 0.95

Coefficients:
            Value    Std. Error t value  Pr(>|t|)
(Intercept)  8.51201  0.36175   23.52998  0.00000
u           -0.23135  0.03801   -6.08665  0.00000
v           -0.20719  0.02989   -6.93281  0.00000
tke          0.02528  0.00637    3.97136  0.00007
tailw        0.38449  0.04350    8.83862  0.00000

Final model: u+v+tke+tailw


tau: [1] 0.99

Coefficients:
            Value    Std. Error t value  Pr(>|t|)
(Intercept) 12.27170  1.01277   12.11696  0.00000
u           -0.09077  0.08987   -1.00998  0.31263
v           -0.08601  0.05424   -1.58590  0.11293
tke          0.00550  0.01110    0.49576  0.62012
tailw        0.48670  0.05780    8.42052  0.00000

Remove term: tke



Step 3


tau: [1] 0.5

Coefficients:
            Value    Std. Error t value  Pr(>|t|)
(Intercept)  0.11496  0.01643    6.99884  0.00000
u           -0.00185  0.00203   -0.91076  0.36254
w            0.12041  0.09538    1.26243  0.20695
tailw        0.00356  0.00130    2.73595  0.00628

Remove term: u-wind
```

```
tau: [1] 0.6

Coefficients:
            Value    Std. Error t value  Pr(>|t|)
(Intercept)  0.24962  0.02977    8.38575  0.00000
u           -0.00491  0.00304   -1.61758  0.10592
w            0.25380  0.14778    1.71739  0.08607
tailw        0.00517  0.00182    2.83723  0.00460

Remove term: u-wind


tau: [1] 0.7

Coefficients:
            Value    Std. Error t value  Pr(>|t|)
(Intercept)  0.52172  0.05151   10.12936  0.00000
u           -0.01690  0.00536   -3.15198  0.00165
w            0.50696  0.23836    2.12688  0.03356
tailw        0.00784  0.00285    2.75260  0.00597

Final model: u+w+tailw


tau: [1] 0.8

Coefficients:
            Value    Std. Error t value  Pr(>|t|)
(Intercept)  1.19713  0.16760    7.14257  0.00000
u           -0.03741  0.02039   -1.83497  0.06667
v           -0.02421  0.01280   -1.89106  0.05877
tailw        0.01536  0.00918    1.67389  0.09432

Final model: u+v+tailw


tau: [1] 0.99

Coefficients:
            Value    Std. Error t value  Pr(>|t|)
(Intercept) 12.62034  0.45149   27.95292  0.00000
u           -0.08899  0.03526   -2.52422  0.01168
v           -0.06746  0.03409   -1.97872  0.04799
tailw        0.45406  0.03324   13.66128  0.00000

Final model: u+v+tailw
```

```
Step 4


tau: [1] 0.5

Coefficients:
            Value   Std. Error t value Pr(>|t|)
(Intercept) 0.10136 0.01020    9.94116 0.00000
w           0.14217 0.08379    1.69672 0.08991
tailw       0.00396 0.00128    3.09818 0.00198

Final model: w+tailw


tau: [1] 0.6

Coefficients:
            Value    Std. Error t value  Pr(>|t|)
(Intercept)  0.21785  0.01803   12.08039  0.00000
w            0.32768  0.16947    1.93358  0.05331
tailw        0.00528  0.00231    2.28414  0.02247

Final model: w+tailw
```

## Results

```
MODELS (by quantile):

0.5: speed ~ w + tailw
0.6: speed ~ w + tailw
0.7: speed ~ u + w + tailw
0.8: speed ~ u + v + tailw
0.9: speed ~ u + v + tke + tailw
0.95: speed ~ u + v + tke + tailw
0.99: speed ~ u + v + tailw
```
